# Patient perspectives on digital patient reported outcomes in routine care of inflammatory bowel disease

**DOI:** 10.1186/s41687-021-00366-2

**Published:** 2021-09-17

**Authors:** Amalie Søgaard Nielsen, Charlotte W. Appel, Birgit Furstrand Larsen, Lars Kayser, Lisa Hanna

**Affiliations:** 1 Diagnostic Centre, University Research Clinic for Innovative Patient Pathways, Silkeborg Regional Hospital, Aarhus University, Aarhus, Denmark; 2 Section of Health Service Research, Department of Public Health, University of Copenhagen, Øster Farimagsgade 5, 1014 Copenhagen, Denmark; 3 School of Health and Social Development, Deakin University, Geelong, Australia

**Keywords:** Digital patient reported outcomes, Digital health, eHealth

## Abstract

**Background:**

Digital patient reported outcomes are used increasingly in daily care and treatment of inflammatory bowel disease. Their purpose includes increased focus on patient wellbeing, reduction in avoidable follow-up consultations and increased patient self-management. However, implementation issues occur and studies indicate patients may have concerns, particularly regarding having fewer face-to-face consultations. This study aims to explore patients’ perspectives of use and non-use of digital patient reported outcomes and to understand the mechanisms underpinning patient reluctance to engage with this health technology.

**Results:**

Sixteen patients with inflammatory bowel disease at a regional hospital in Denmark were interviewed about their experiences of, and perspectives on, digital patient reported outcomes. A certain level of eHealth literacy was found to be a fundamental condition for use, while other factors were barriers or facilitators for use of digital PROs. Patients’ main concerns were about potential consequences for their care and relationship with the clinic. Most patients in stable remission were satisfied with the hospital being a “life-line” if their symptoms worsened, and perceived digital patient reported outcomes to be an efficient tool to establish that “life-line”. Patients with severe symptoms and a high degree of emotional distress related to their disease valued the potential for digital patient reported outcomes to increase their clinicians’ focus on mental health and extra-intestinal symptoms.

**Conclusion:**

This study found that if patients had sufficient digital literacy, they perceived digital patient reported outcomes to be a useful replacement for face-to-face consultations. However, they were concerned about digital patient reported outcomes’ effect on the patient–clinician relationship and its ability to detect worsening of symptoms. These concerns may be mitigated by good patient–clinician relationships, and the option for patients to maintain direct telephone contact with their gastroenterology specialist.

**Supplementary Information:**

The online version contains supplementary material available at 10.1186/s41687-021-00366-2.

## Plain English summary

‘Digital patient reported outcomes’ refer to systems by which health professionals collect health information from patients between consultations, mostly via self-completed online questionnaires. This approach aims to support treatment and disease management, and reduce avoidable face-to-face consultations between doctor and patient. However, patients may have concerns about using digital systems to communicate with their clinicians, particularly regarding having fewer face-to-face consultations. To find out more, we interviewed sixteen patients at an Inflammatory Bowel Disease outpatient clinic in Denmark, where digital Patient Reported Outcomes have been used since 2017. We found out that patients needed to have enough familiarity with technology to be able to use these online systems. Patients were worried about how this new way of communicating with the clinic affected their care and their relationship with their doctors and nurses. People with Inflammatory Bowel Disease can have long periods of time with very little disease activity, and in these stable periods, a lot of patients found that a digital Patient Reported Outcomes system was a good way to stay in touch with the clinic and keep reporting their symptoms online– as long as they still had the possibility of contacting the clinic by telephone if needed. During disease flare-ups, patients believed that digital patient reported outcomes’ could be used to give broader insight into their health situation, and trusted their clinicians to use their self-reported data to improve their care.

**Supplementary Information:**

The online version contains supplementary material available at 10.1186/s41687-021-00366-2.

## Background

Patient reported outcomes (PROs) are defined as “any report coming directly from the patient about a health condition and its treatment” [1]. PROs are increasingly becoming a part of routine health care services for a wide range of long term conditions [2, 3]. The Danish Health Data Board has initiated national implementation of PRO data in the clinical care of a broad range of chronic conditions [4]. In addition, Central Region Denmark implemented digital PROs by the use of the generic platform Ambuflex for multiple chronic conditions. One of these implementation sites was the outpatient clinic for chronic inflammatory bowel disease (IBD) at Diagnostic Centre, University Research Clinic for Innovative Patient Pathways, Silkeborg Regional Hospital (DC). At this site, digital PRO data are used in various ways: as a clinical follow-up by replacing a face-to-face consultation; by clinicians and patients in preparing for the consultation; to detect symptom exacerbation between consultations; and to prompt patients to contact the clinic.

IBD is a common term for the diseases Ulcerative Colitis (UC) and Crohn’s disease (CD) which are chronic, lifelong gastrointestinal disorders. Each person with IBD has significant variation in their pattern and severity of symptoms. There is currently no cure for the disease, but in most cases it can be managed efficiently with the use of medications and clinical follow up visits [5]. The chronic nature of the disorder, including a pattern of multiple relapses, can have wide-ranging influences on a person’s emotional, physical, sexual and social wellbeing [6, 7]. In addition, chronic stress and depression symptoms are believed to affect relapse [8].

Reviews suggest that IBD self-management interventions can improve health and well-being [9, 10]. Self-management can be promoted by the use of digital PRO questionnaires [3, 10]. Digital PROs are in this case understood as digitally-administered questionnaires surveying an individual’s health and well-being in a clinical context [11].

Despite the multiple benefits of using PROs (digitally administered or otherwise) in clinical practice, barriers to implementation occur [12–14]. Some of these barriers can be explained by divergent patient perspectives on the use of PROs—especially regarding replacement of face-to-face consultations [15]. Studies have shown that people with IBD are willing to participate in digital PRO systems [16, 17]. However, patients have different needs for care and follow-up [18] and might have concerns about using digital PROs that could lead to barriers to implementation or dropout [15]. IBD patients’ perspectives on digital PROs have not been widely studied. Therefore, the aim of this study was to explore IBD patients’ experiences of, and perspectives on, an implemented digital PRO system.

## Method

This paper reports on qualitative interviews with IBD patients as one component of a larger mixed-methods action research based study of users and non-users of digital PROs.

### Setting

The study was conducted at a regional Danish hospital’s outpatient IBD clinic, where digital PRO data have been used in a range of ways in routine practice since 2017. Since then, any patient with a national secure email (95% of the general population) and with adequate fluency in Danish has been eligible to opt-in to using the digital PRO system *Ambuflex* when asked by their hospital clinician. When this study was conducted, 77% of the IBD clinic’s patients were enrolled in the digital PRO system*.* The PRO questionnaire was developed in an iterative process by clinicians and patients at the clinic, and consists of 44 questions addressing intestinal and extra-intestinal symptoms (including the Harvey-Bradshaw Index and the Simple Clinical Colitis Activity Index [19, 20]), health-related quality of life, and need for patient–clinician contact.

### Sampling and recruitment

To ensure a broad sampling participants were recruited purposively based on their responses to a survey sent to all patients at the clinic (Table 1) as part of the wider study. The survey contained the Readiness and Enablement Index for Health Technology (ReadHy) questionnaire, which is validated in a Danish hospital context [21] exploring individuals’ prerequisites to engage in digital health interventions, building on the eHealth Literacy Questionnaire [22] and including scales from the Health Literacy Questionnaire [23] and the Health Education Impact Questionnaire [24]. Responses to the ReadHy questionnaire were the foundation for a cluster analysis that grouped the patients into six groups representing their eHealth readiness. Representatives from each group were invited to participate in a qualitative interview. Eight women and eight men (29–63 years old), four with Crohn’s disease and 12 with Ulcerative Colitis, were recruited. Fifteen of the sixteen participants were enrolled in the PRO system at the IBD clinic (Table 1).

**Table 1:** Interview participants divided into groups based on their responses to the ReadHy questionnaire (Gr1-Gr6)

Group	Age	Gender	Diagnosis	Mode	PRO
Gr1	73	Male	CD	Telephone	No
Gr2	39	Male	UC	Hospital	Yes
Gr2	53	Female	CD	Telephone	Yes
Gr3	32	Female	UC	Hospital	Yes
Gr3	40	Female	UC	Hospital	Yes
Gr3	33	Male	UC	Hospital	Yes
Gr3	43	Female	UC	Telephone	Yes
Gr4	44	Female	CD	Home	Yes
Gr4	58	Female	UC	Home	Yes
Gr4	61	Male	UC	Home	Yes
Gr5	64	Female	UC	Hospital	Yes
Gr5	46	Male	CD	Hospital	Yes
Gr5	53	Female	UC	Telephone	Yes
Gr6	29	Male	UC	Hospital	Yes
Gr6	65	Male	UC	Telephone	Yes
Gr6	57	Male	UC	Home	Yes

*UC* ulcerative colitis, *CD* Crohn’s disease

### Data collection

Individual, semi-structured qualitative interviews were held with 16 participants in April–August 2019. The first author, who had no prior relationship to the participants and no health professional background, conducted the interviews using a semi-structured topic guide (Additional file 1) [25] to explore patients’ perspective on digital PROs. Follow-up questions, exploratory questions and interpretative questions were used [25]. Interview duration varied from 28 to 66 min, with a mean duration of 42 min.

Interviews were conducted at the hospital (n = 7), in the patients’ home (n = 4), by phone (n = 5), or in a public space (n = 1) in accordance with participants’ wishes (Table 1). Interviews were recorded and transcribed verbatim.

### Data analysis

**Fig. 1:**
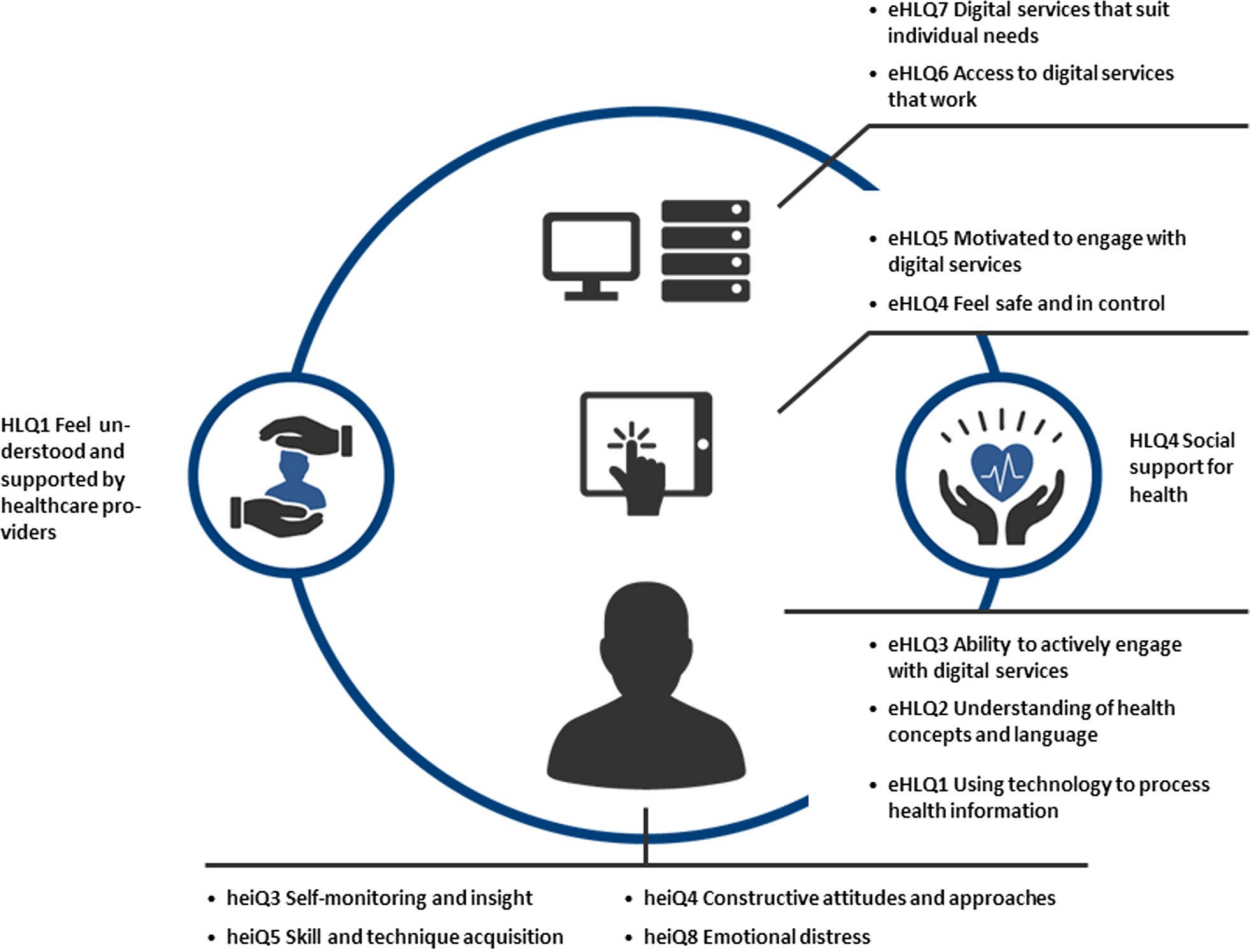
The ReadHy framework. Reproduced under ©creative commons from journal of medical internet research (http://www.jmir.org), 12.02.2019

An abductive approach was used for data analysis [26]. First, the interviewer listened to the interview recordings and noted some general themes. Afterwards, the transcripts were read through, and open coding was conducted by the interviewer using NVivo software. These codes were categorized and grouped into initial themes by the interviewer and the last author. Finally, these themes were analysed deductively using the theoretical foundations from the ReadHy framework [21], and other relevant literature [26]. The ReadHy framework consists of 13 dimensions (Self monitoring and insight, Constructive attitudes and approaches, Skills and Technique Acquisition, Emotional distress, Feeling understood and supported by healthcare providers, Social support for health, Using technology to process health information, Understanding of health concepts and language, Ability to actively engage with digital services, Feel safe and in control, Motivated to engage with digital services, Access to digital services that work, Digital services that suit individual needs), which together offer insights into users’ readiness towards health technology, shown in Fig. 1. Using this theoretical framework focus is on abilities, emotional states and contextual factors.

### Findings

Several themes were generated based on the interview data. Each theme was regarded a benefit or concern (or both) regarding use of digital PROs and mapped to dimensions from the ReadHy framework (see Table 2).

**Table 2:** Themes in interviews with IBD patients—grouped into ReadHy dimensions

Dimension	Benefit	Concern
Digital services that suit individual needs	Increased patient convenience	Not fit for smartphone
		Unused potential of the system
Motivated to engage with digital services	Holistic care	Miss updates from hospital
	Informing consultations	No control on next appointment—being lost in the system
Feel understood and supported by healthcare providers	Prioritising hospital resources	Fear of being forgotton in the system
		Need for “life-line”
Feel safe and in control	Data is used in proper way	Is data used in proper way?
Understanding of health concepts and language	Learning from the PRO questions	Difficulties with free text field
Emotional distress	Compare with others	Compare with others
		Increased anxiety
Self-monitoring and insight		Hard to assess symptoms
Ability to actively engage with digital services		Need to be able to use computers or smartphone

### Primary reasons to engage

Most participants reported that their primary motivation for using the digital PRO system was that the hospital had asked them to. They therefore seemed to be motivated by external rather than internal factors [27]. Participants reported a high degree of trust in, and a good relationship with, the clinic. For example:

“You got to trust it. I think I do that, and it is only because I hope… this department has always been good… I hope it is still really good.” Male, 46

Despite this external motivation for its use, many participants elaborated further on why they thought digital PROs was a positive addition to their care. All participants, regardless of whether they had used the PRO system, reported that they thought it was beneficial for their current situation, reflecting the ReadHy framework dimension of *motivated to engage with digital services.* These benefits are elaborated below.

### Perceived benefits of digital PROs

#### Increased patient convenience

Some participants felt that avoiding hospital appointments (for example when asymptomatic) was the main benefit of the digital PRO system:

“I agreed to participate in that AmbuFlex-thing, because I just get ill when I come in here [at the hospital]. I’m not sick, why should I then sit up there talking to a doctor, saying, are you all right? Yes I’m all right.” Male, 33 years.

“It fits me well that I do not have to drive 100 km back and forth to go to the hospital. That being as long as everything is as it is right now.” Female, 53 years.

The convenience of not spending time visiting the hospital, when perceived to be unnecessary, suited participants’ individual needs. However, it was important to participants to be able to visit the clinic when they perceived it to be necessary. However, it also reflects potential patient worries, which will be described in more detail subsequently.

#### Holistic care, informed consultation, and proper use of data

Some participants always preferred to attend the clinic physically, regardless of inconvenience:

“Maybe it is very troublesome to drive in there, but I prefer the personal conversation with real people.” Male, 57 years.

In this case, the participant still received the PRO questionnaires, but used them as a preparation for consultation, even though he only visited the clinic once a year; this is an example of the individually-tailored use of digital PROs that had been implemented in this setting. However, most participants identified that using digital PROs informed and enhanced their face-to-face consultations, making them more efficient:

“I guess it is to get the information, to be prepared and to get a holistic view—also backwards to see, if there is anything to see, when you get enough questionnaires filled out”, Male, 61 years.

Again, patients trusted that clinicians read their PRO data before a consultation, reflecting that participants felt *safe and in control* of their data:

“I do not experience that they ask about the things I wrote […] But that I assume. I don’t know, of course, I don’t ask if they have read it, but that I assume”. Male, 60 years.

In addition, some of the patients who used the digital PRO system for follow-up and replacement of consultations also found beneficial the inclusion of questions on issues like mental health, fatigue and sexual function:

“many of the consultations you encounter are based on blood samples, and then you just talk about that and not so much about me, who is sitting over here. Then it can be… then these questions come… but ok, they actually do ask this in these questionnaires. But it will never be sufficient to see the entire me. Right?” Male, 57 years.

Some patients were unsure how this additional information was used, for example the patient below who suffered from a great deal of mental health issues in combination with her IBD:

“I did think, maybe they are starting to take these issues seriously. A whole picture of the well-being of the person they actually start to be aware of. And then I think it is positive. But if they do not intend to use it for anything, then of course I think it is not worth much.” Female, 43 years.

The participants felt *motivated to engage with digital services,* due to these different ways in which the digital technology could be used as a means to interact with healthcare providers.

#### Prioritising resources

Participants were asked what they believed to be the hospital’s reason for implementing the digital PRO system. All of the participants who received digital PROs as a replacement for consultations responded that they believed that the primary purpose was to save money by having fewer physical consultations at the hospital. Most of them perceived this to be a good thing, as they thought the hospital should direct their resources either to other patients who needed them more, or to themselves when they felt worse. When asked why she thought the system had been implemented at the hospital, one participant responded:

“To save time, I guess […] It is ok with me […] if it can save some money, then they can use the money when I come in all sick.” Female, 64.

Again, this statement reflects a degree of trust in the clinic and that the participants felt *understood and supported by healthcare providers*. The participants trusted that the money (and time) will be used appropriately on their care when they needed it.

#### Learning from the PROs

Some of the participants highlighted that digital PROs enhanced their knowledge about their disease and *their understanding of health concepts*, which again affected their engagement with their health and healthcare:

“Some of the questions asked in the questionnaire have I never received before through ten years of consultations. […] I just thought; that is how it is. Until that questionnaire came. It is… something about… I think it was sore spots on the skin or something. Like when you touch me, you don’t have bruises, but it just hurts those places, that is also the bowel disease, that I have never thought, I just thought it is because I am a little weak.” Female, 32 years.

### Concerns about digital PROs

Even though participants accepted digital PROs and perceived there to be individual benefits in engaging with these systems, they still expressed concerns regarding their use. These themes are presented below as dimensions of the ReadHy framework: *feeling supported by healthcare providers; emotional distress, self-monitoring and insight,* and *motivated to engage with digital services*.

#### Need for “life-line”

Participants agreed that using digital PRO systems is appropriate when their IBD was less symptomatic, but did not perceive digital PROs to be useful when their symptoms were severe. It was important to participants that digital PROs were not used as a replacement for synchronous or face-to-face consultations if needed. A need for supplementary support from healthcare providers was expressed:

“first I thought, well, you know your life line sort of disappears, but then again, I know the phone is there.” Female, 40 years.

In this case, the participants perceived their ‘*life-line*’ to be direct support from their healthcare provider. Even when in stable remission, participants talked about their fear of not having the support in case of flare-ups.

#### Increased anxiety

Participants found that being asked the same questions repeatedly via digital PROs caused both concern and reassurance. Some participants found that their anxiety increased when they were asked about symptoms they did not have, but might expect to get:

“Well… there are some symptoms I have never had. They just keep popping up again and again in the questionnaire. But then again it is not targeted me, it is targeted for a group of patients. Then the answer is simple, I guess, but it is that thing about, when you have never had that symptom before, then maybe you have had without knowing. But I don’t think I have, but you… it makes you… That’s the hard part, well, now I say the hard part, it is also where you are grateful, that there are symptoms that you do not have.” Female, 40 years.

Therefore, patients’ level of emotional distress with regard to their health situation may be affected by the digital PROs, often in a negative direction, but sometimes the comparison makes you “grateful” of the symptoms you do not have.

#### Hard to assess symptoms and difficulties with free text field

Participants in this study found it difficult to rate their own pain levels, particularly over time:

“I do not think it is easy… but I do answer of course. Everything is… It does not hurt as much so you’re not able to walk…. And then you think… how do you modulate that… I’m able to go to work, I’m able to walk… It is not like that it affects me in a way where I’m not able to work. I imagine others feeling that way… so if I have to modulate it, then my score is rather low. Also compared to how you can feel…. When I was sick.” Female, 53 years.

This reflects the ‘Self-monitoring and insight’ dimension of the ReadHy framework reporting on patients’ ability to continuously assess their symptoms and monitor the development of their disease, and includes patients’ knowledge about how to respond appropriately to symptom changes.

In addition, one participant reported cognitive difficulties affecting her recall ability:

“Then they ask ‘how have you been the last year?’, and then I have to sit there and say I do not remember. I only remember… I’m very in the moment… and maybe last week and then not much longer. I do not remember how I felt a month ago or how I was.” Female, 43 years.

The PRO system included a free text field, which challenged patients who found it difficult to articulate information about their health in writing, reflecting a deficit in the ReadHy dimension of *understanding of health concepts and language*. When asked what he would do if he was experiencing a symptom flare-up, this participant responded:

“I would prefer talking to a doctor. Most likely. Because there is always something, some questions, some thoughts, that, when you sit in front in a consultation, will appear. I think it is like that, if you have a comment or something in the end of the questionnaire, you do not always get it written, and how are you supposed to write something like that. It is difficult to express feelings. It is easier to look someone in the eye.” Male, 33 years.

In addition, two participants reported they were dyslexic, which meant that they found it more difficult to use a free text field:

“I’m not good at… I’m dyslexic, I’m not good at formulating thing in writing. I just like to check boxes, and that’s really fine, that you are supposed to do that in the questionnaire.” Male, 46 years.

Most of the other participants also reported that they did not use the free text field often.

#### Being lost and missing updates

Some participants had concerns about being lost in the system after being assigned to the digital PRO system, therefore perceiving the digital PROs as a barrier to interaction with their healthcare providers. These patients reported that they usually left the hospital with a confirmed follow-up appointment in 12 months’ time, but when using digital PROs they were not clear when they would receive their next appointment:

“It took a while before I got one [a PRO questionnaire]. It is almost… I think it is a year after we discussed it, that I got one. Why, I don’t know. But then again, they had not promised that it would be fast. But it took a long time. I did come to think I was forgotten.” Male, 46 years.

A change in the organisational setup has been made after this initial finding, making sure that the patients receive the first PRO questionnaire right after the first appointment.

In addition, some participants found it rewarding to get news updates from the doctor during face-to-face consultations. These could be about a new type of medicine, medical trials or new information about their disease. Disease control was very important for many participants, and their clinicians were the most trusted source of information about treatment:

“I do know that [doctor’s name] at some point had an idea that I was supposed to enter some sort of combo-treatment something, but then you have to have 20 cm of inflammation and I did not […] I don’t know if they think of something like that when you just—in quotation mark—is in Ambuflex. So that part is… That’s why I still think it is important to see your doctor once in a while. Maybe every other year or so.” Female, 40 years.

This reflects whether patients perceive digital technology to have a positive impact on how they can manage their health and interact with healthcare providers; ReadHy dimension ‘Motivated to engage with digital services’. In this study, patients raised concerns about technology making disease management and communication more difficult.

#### Need to be able to use a computer or smartphone

None of the participants who were assigned to use digital PROs reported any difficulties in using the digital system, indicating high *ability to actively engage with digital services*. However, there were some difficulties in achieving *Digital Services that suits individual needs*; one participant had tried to complete the questionnaire on a smartphone and did not find it suitable:

“I did actually sit down with my phone and did it. That’s a bad idea. It is actually quite difficult to read it then, because you all the time have to scroll the page from side to side to be able to read, I’ll say it is a good idea to be on the computer next”. Female, 32 years

Other participants reported using their computer to complete the PRO questionnaire, which reflected their need to concentrate when doing so:

“It is not something I just do. I do take it seriously, and I make sure that I’m in a closed environment, that I’m able to have 100% focus, because it is after all my gut. It is not just for some sort of statistic…” Female, 40 years

The one participant who did not use digital PROs reported that he did not know how to use computers, even though he used mobile phones and hands-free electronic devices. He had concerns regarding *ability to actively engage with digital services*. He emphasised that he would not use a digital PRO system, but did share that that he would like himself and the doctor to know more about his disease and that he was heavily affected by his illness:

“I do not think I gain anything from it [talking to the healthcare professionals]. I do not think they can tell me anything […]. It [the disease] is something I live with, and I will never get over it, so of course it is to my benefit to know as much as possible.” Male, 73 years

#### Unused potential of the system

While few participants had trouble with the digital aspect of the digital PRO system, others would have liked it to be even more advanced in order to quantify their disease status and serve their individual needs:

“If I compare with my stepfather, he got diabetes, one tool he got is that he can pierce himself in his finger and then he takes this glucose test, and it helps him to adjust his medicine, and it gives him an idea of how he is feeling, if he cannot feel it otherwise. I can’t do that. I wish I could. Then I all the time have to feel. I’m bad at that”. Female, 40 years

However, some participants were reluctant to use the questionnaire as a self-service, because they knew that nurses would check their answers and react on them:

“If you could go in and pull out some charts. You can’t use it like that. As soon as you go in and fill out a questionnaire, then a larger process is starting where someone has to sit and read the questionnaire and assess it… if it is one thing or another. But I do like charts.” Male, 39 years.

## Discussion

This study supports a growing body of evidence, both in Denmark and globally, on the use of digital PROs. It presents the perspectives of 16 people with IBD attending a specialist clinic with a well-implemented digital PRO system. Patient perspectives were analysed through a the ReadHy framework of eHealth Literacy and Readiness. The ReadHy framework focuses on different aspects of patient–clinician interaction, on patient competence and attitudes, and on contextual surroundings [21]. The deductive component of our analysis used this framework, and found concerns and benefits within the dimensions *Self-monitoring and insight*, *Emotional distress*, *Feeling understood and supported by healthcare providers, Understanding of health concepts and language*, *Feel safe and in control*, *Motivated to engage with digital services,* and *Digital services that suit individual needs*. A single participant raised concerns regarding A*bility to actively engage with digital services.* The remaining dimensions of ReadHReadHy *(Constructive attitudes and approaches, Skills and Technique Acquisition, Social support for health, Using technology to process health information, Access to digital services that work)* were not represented in the data. This could be a result of a limited focus from the interviewer. Another reason could be that the patients did not pay attention to questions within these dimensions and therefore no conclusions should be drawn on behalf of these “missing” dimensions.

Following the ReadHy framework, this study indicates that high uptake of digital PRO systems is partly attributable to patients feeling supported and understood by their healthcare providers. In this study’s clinical setting, patients were introduced to the digital PRO by the clinicians, often by a doctor in the first instance during a face-to-face consultation, with follow-up after the consultation by a nurse. This study’s participants’ perspectives on the digital PRO solution were influenced by these encounters with clinicians, and participants’ reasons for uptake was often that “the hospital asked”; all had accepted enrolment in the digital PRO system when asked. The one patient who was not enrolled was never asked. This finding corroborates prior research showing that trust in technology and trust in clinicians are linked [28].

Participants had varied perspectives on how the system fitted or did not fit their individual needs, which aligns with the ReadHy framework [21] as well as motivational theories [27, 29] which emphasise the perceived purpose and benefit of the system to its user. The most obvious benefit of the system to participants, as with many eHealth systems, was that they did not have to spend time visiting the hospital when they felt it was avoidable [15, 30]. The changing nature of IBD is essential here, as patients may easily experience long periods of very low disease activity, which for some participants meant they did not feel like patients at all, whilst others still remained very conscious of their disease, indicating different needs for care [18].

All participants acknowledged that the hospital may use the PRO system to prioritise resources and were comfortable with that process. This finding might differ in other settings, as in this study it was dependent on participants’ trust that if the hospital saved money on avoidable consultations, this money would be spent on enhancing care elsewhere in their patient pathway.

On the other hand, the reduction in consultation frequency did lead to some concerns among the participants in this study; this might influence dropout [31], or explain why the uptake of digital PROs in the study setting was 77% rather than all eligible patients. Some participants in this study simply preferred physically meeting with their doctor. A face-to-face consultation was perceived as the “gold standard” for communication and some patients did not value saving time, but preferred to that everything remained as usual [32]. In these circumstances, digital PRO systems may still function as preparation for the face-to-face consultation rather than a replacement, and tap into the desire by patients for stronger clinician-patient communication that has been found in other studies [33]. Other patients worried about being forgotten within the digital PRO system. This concern has also been found in a similar study of PROs as follow-up for people with epilepsy [15].

The fear of being forgotten relates to the patient’s relationship with their healthcare provider and their trust in their ability to access health care. All participants in this study were confident they could call the clinic if their condition worsened; telephone calls were widely used and maintained a strong communicative relationship with the clinic despite the decrease in consultations as a consequence of digital PROs. Increased telephone contact from patients to IBD clinics has been shown to correlate with increased severity of the disease and its impact on patients’ daily life [34].

Another concern related to the decrease in consultation frequency was patients’ desire to be updated on developments in IBD research and to be invited to participate in research projects studying new interventions. This relates to some participants’ need to increase their knowledge about their disease and health status. This study’s participants did not feel digital PROs could address this need. Many of the participants expressed frustration that they, as well as their healthcare professionals, did not know why their disease developed, how they could control their disease, or how it could be cured. This frustration has also been found in prior studies of unmet needs amongst patients with IBD [33, 35]. Information on disease management can be difficult to disseminate through digital PROs. However, this is an obvious area of possible improvement of the system and should be taken into account in future implementations of digital PROs as follow-up.

Additionally, some participants in this study worried whether digital PROs were able to capture a worsening in their symptoms, and found it hard to report on their symptoms at each time point. Participants’ relationship with the clinic was important here too. Most participants trusted that there was a medical reason underpinning the digital PRO questions, but some found it hard to answer the questions on their own experiences of their health and well-being. This is corroborated by other PRO studies [15]. Also, research suggests that patients with low health literacy might have more difficulty answering questions on symptoms and find doing so more worrying [36]. Participants in this study tended not to use the PRO questionnaire’s free text field to provide further information. These difficulties could lead to patient frustration with digital PROs and increase patient concern about their ability to manage their own health, which is believed to be important in patient acceptance of digital health interventions [21, 37]. Issues like these could be addressed in any introduction to a digital PRO system that clinicians provide to patients. To support patients’ confidence in using digital PRO, this introduction might need to focus more on the content of the questionnaire, how to complete it and how patient-reported data are used.

Participants in this study differed in how much their illness disturbed their daily life. Some were relatively unaffected, whilst others were heavily affected and had lost their job or become psychologically distressed or socially isolated. Studies have shown that mental health issues occur commonly amongst patients with IBD, especially during periods of disease activity compared to remission [38], and that mental health issues are known to affect treatment adherence [39]. Digital PRO systems offer a means for patients to share information on mental health and health-related topics with their clinicians. Participants in this study appreciated this functionality; it was believed to be one of the main benefits of the digital system and of great importance to the patients. The increased focus on psychological aspects of the disease has also been found in other studies of PROs [40–42]. In addition, the digital PRO’s focus on extra-intestinal symptoms was found to lead to a small increase in health literacy among some of the participants, which could support self-management [43]. However, patients’ increased awareness of symptoms, both regarding mental health, abdominal pain and others, had some negative aspects. Some participants experienced increased anxiety when confronted with possible consequences of their condition.

Overall, in the study setting the individualised fitting of the system and the patient–clinician relationship functioned as facilitators for the use of digital PROs. The barriers for its use were the difficulties of self-monitoring symptomatology, potential for increased emotional distress, the perception that digital PROs reduce disease control and communication with clinicians, and fear of being forgotten by healthcare providers. These concerns should be explicitly addressed with patients by clinicians during implementation of digital PROs. In contrast, the technology aspect of digital PRO was not a concern for most of the participants. Some participants had a higher need for self-monitoring, and asked for additional features in the system, but none complained that it was difficult to use. Despite this, the one participant who had not enrolled in the digital PRO system attributed this to the technological aspect of doing so, indicating that the digital aspect is very fundamental, and has to be accepted prior to most other aspects.

### Strengths and limitations

This study used a specific framework- the ReadHy framework [21]—in the analysis of qualitative interview data. Using an a priori framework, and the concepts it contains, limits the scope of the analysis, but also focusses it on the complex mechanisms believed to be present when patients interact with eHealth.

A limitation to this study is its recruitment of only one patient who was not using the digital PRO system. Ideally the study would have recruited participants who had actively declined uptake of the digital PRO system, or had enrolled in the system but subsequently dropped out, to capture their perspectives on reasons for non-use. However, using participants’ responses to the ReadHy questionnaire (collected via the prior quantitative component of this study) in recruitment enabled a broad representation of patients across different levels of eHealth readiness, and this strengthens the results. The concerns of patients using the intervention may shed light on non-users’ potential concerns.

## Conclusion

This study found that as long as patients were comfortable with the technological aspect of a digital PRO system, they found it a reasonable means to reduce avoidable face-to-face consultations and were therefore willing to accept its use. However, the participants had some concerns about whether the system was good enough to detect worsening in symptoms and if a problem would go undetected between reduced face-to-face consultations. Patient concerns of this nature may affect their willingness to use the digital PRO, but can be addressed by a good clinical relationship and the importance of direct telephone contact between patient and clinician if needed. This knowledge is important for the design of new digital PRO systems.

This study showed that participants did trust that resources saved by the use of digital PROs may be redistributed to improve clinical treatment for themselves and others. This trust is fundamentally connected to patients’ relationship to clinicians and to the structure of the wider healthcare system. Overall, this study illustrates the importance of the patient–clinician relationship when implementing digital PROs.

## Supplementary Information

**Additional file 1**. Interview guide.

## Data Availability

Data is not freely available due to the privacy of participants, but parts of data may be released in anonymous form. Please contact corresponding author.
