# Supplementary material for: Patient perspectives on digital patient reported outcomes in routine care of inflammatory bowel disease
Source: J Patient Rep Outcomes. 2021 Sep 17;5:92. doi: 10.1186/s41687-021-00366-2 (PMC8448812; doi:10.1186/s41687-021-00366-2)
Supplement: Supplementary file 1 — Additional file 1. Interview guide. [file 41687_2021_366_MOESM1_ESM.docx]

Interview guide –translated into English

Try to tell me about the first time you were introduced to AmbuFlex / IBD

- How were you introduced to the system?

- What did you think about the questionnaire itself?

- What do you think about the questions that are being asked?

- Why do you think you have been introduced AmbuFlex / IBD here at the department?

- Is there anything about AmbuFlex that you think is good?

- Is something you think is bad?

Try to tell me how you usually fill in the questionnaire?

- When do you fill in?

- Where do you fill in?

- How long do you spend approx. on it?

- Is it easy or difficult? Why?

- How do you experience the severity of your symptoms?

Does the questionnaire affect how you think about your illness?

- Do you often think about your illness?

Does the questionnaire change the actions you do in relation to your illness / health?

Do you learn anything from filling in the form?

Try to tell me about the last time you were in contact with the department

- Who did you talk to?

- Did you fill out the questionnaire in advance?

- Did you talk about the questionnaire during the consultation?

- How did you experience it?

- Has the questionnaire changed the way you talk to your doctor or nurse? Can you give some examples?

Do you feel safe, when using AmbuFlex from home?

- Are you happy to use a computer to fill out the questionnaire?

- Is there anything that worries you?

What do you think about AmbuFlex in general?
